# Supplementary material for: Representation Retrieval Learning for Heterogeneous Data Integration
Source: J Am Stat Assoc. Author manuscript; Available in PMC 2026 Oct 8. (PMC13645369; doi:10.1080/01621459.2026.2696485)
Supplement: Supplementary file 1 [file NIHMS2211748-supplement-Supp_1.zip › uasa_a_2696485_sm7980.docx]

Author Contributions Checklist Form

This form documents the artifacts associated with the article (i.e., the data and code supporting the computational findings) and describes how to reproduce the findings.

# Part 1: Data

☐ This paper **does not** involve analysis of external data (i.e., no data are used or the only data are generated by the authors via simulation in their code).

☒ I certify that the author(s) of the manuscript have legitimate access to and permission to use the data used in this manuscript.

## Abstract

In this paper, we analyzed two real-world datasets. One is pan-cancer data, including five types of cancer tumors from patient-derived xenograft, and fully-observed three modalities: DNA copy number, RNA count and mutations. The response is the tumor shrinkage after applying certain treatment. In our analysis, we build up predictor to predict the response given all three modalities. The other data is ADNI dataset, including four sources of datasets and blockwise missing modalities: Gene expression, PET, and MRI. The response is MMSE, measuring the mental impairment. We also build a predictor to predict patients’ mental impairment, with partially observed modalities.

## Availability

☒ Data **are** publicly available

☐ Data **cannot be made** publicly available

If the data are publicly available, see the *Publicly available data* section. Otherwise, see the *Non-publicly available dat*a section, below.

### Publicly available data

☒ Data are available online at:

☒ Data are available as part of the paper’s supplementary material.

☒ Data are publicly available by request, following the process described here:

Pan-cancer data is available from https://www.tandfonline.com/doi/full/10.1080/01621459.2020.1828091

ADNI data is accessible from <https://adni.loni.usc.edu/data-samples/adni-data/>. We use the preprocessing code from the paper: <https://www.tandfonline.com/doi/full/10.1080/01621459.2020.1751176>

All processed dataset is included in our supplementary material

☐ Data are or will be made available through some other mechanism, described here:

### Non-publicly available data

Discussion of lack of publicly available data:

## Description

### File format(s)

☒ CSV or other plain text:

☒ Software-specific binary format (.Rda, Python pickle, etc.):

☐ Standardized binary format (e.g., netCDF, HDF5, etc.):

☐ Other (described here):

### Data dictionary

☐ Provided by the authors in the following file(s):

☒ Data file(s) is (are) self-describiing (e.g., netCDF files)

☐ Available at the following URL:

### Additional information (optional)

# Part 2: Code

## Abstract

Our code mainly contains three components:

1. R2_reproduce: include the codebase of our proposed method, script to run the simulation and real data analysis.
2. Competing_reproduce: include the codebase of all competing method, script to run the simulation and real data analysis

3. Combine_results: combine the results from above two folders, and produce figures and tables presented in the paper.

## Description

### Code format(s)

☒ Script files

☒ R ☒ Python ☐ Matlab

☐ Other:

☐ Package

☐ R ☐ Python ☐ MATLAB toolbox

☐ Other:

☐ Reproducible report

☐ R Markdown ☐ Jupyter notebook

☐ Other:

☐ Shell script

☐ Other (described here):

### **Supporting software requirements**

Version of primary software used

Python: 3.12.1

R: 4.4.1

Libraries and dependencies used by the code

Torch: 2.5.0.dev20240808

Numpy: 1.26.4

Matplotlib: 3.8.4

Seaborn: 0.13.2

Proxtorch: 0.0.9

Optuna: 4.2.0

Ggplot2: 4.0.0

### Supporting system/hardware requirements (optional)

Our numerical experiments are primarily running on Mac M3Max chip.

### Parallelization used

☐ No parallel code used

☒ Multi-core parallelization on a single machine/node

Number of cores used: 10

☐ Multi-machine/multi-node parallelization

Number of nodes and cores used:

### License

☒ MIT License (default)

☐ BSD

☐ GPL v3.0

☐ Creative Commons

☐ Other (described here):

### Additional information (optional)

# Part 3: Reproducibility workflow

## Scope

The provided workflow reproduces:

☐ Any numbers provided in text in the paper

☒ The computational method(s) presented in the paper (i.e., code is provided that implements the method(s))

☐ All tables and figures in the paper

☒ Selected tables and figures in the paper, as explained and justified here:

Main text:

Table 1

Figure 3, 4, 6

Supplementary:

Table 1

Figure 1-11

## Workflow details

### Location

The workflow is available:

☒ As part of the paper’s supplementary material

☐ In this Git repository:

☐ Other:

### Format(s)

☐ Single master code file

☐ Wrapper (shell) script(s)

☐ Self-contained R Markdown file, Jupyter notebook, or other literate programming approach

☐ Text file (e.g., a readme-style file) that documents workflow

☐ Makefile

☒ Other (more detail in 'Instructions' below)

### Instructions

For our proposed method, please locate at the “r2_reproduce” folder, and run script “run_all_r2.py” with additional options to generate all results by our proposed method, which can be found in the subfolder “output”.

For competing methods, please locate at the “competing_reproduce” folder, and run script “run_all_competing.py” with additional options to generate all results by competing methods, which can be found in the subfolder “output”.

Lastly, locate at the “combine_r2_competing” folder, and run script “combine_results.py” to generate figures and tables presented in the paper.

Expected run-time

Approximate time needed to reproduce the analyses on a standard desktop machine:

☐ <1 minute

☐ 1-10 minutes

☐ 10-60 minutes

☐ 1-8 hours

☒ >8 hours

☐ Not feasible to run on a desktop machine, as described here:

### Additional documentation (optional)

# Notes (optional)
