# Supplementary material for: Representation Retrieval Learning for Heterogeneous Data Integration
Source: J Am Stat Assoc. Author manuscript; Available in PMC 2026 Oct 8. (PMC13645369; doi:10.1080/01621459.2026.2696485)
Supplement: Supplementary file 1 [file NIHMS2211748-supplement-Supp_1.zip › online_supp (1).zip!/online_supp/appendix.pdf]

Appendices of "Representation Retrieval Learning for Heterogeneous Data Integration"

Qi Xu* Annie Qu†

A Multivariate representer output

In this part, we provide a detailed treatment when representers output a multivaraite latent representation. Formally, we consider $\theta_d : \mathcal{X} \to \mathbb{R}^{r_d}$, and the output of the dictionary Θ is of dimension $\sum_{d=1}^{D} r_d$. The corresponding learner can be formulated as $\beta^{(s)} = (\beta_{(1)}^{(s)}, \cdots, \beta_{(D)}^{(S)})$ for $s = 1, \cdots, S$ where $\beta_{(d)}^{(s)} \in \mathbb{R}^{(r_d)}$. The ℓ_1 penalty on learners in Section 3.1 are adapted as ℓ_2 penalty, i.e., group lasso penalty on each $\beta_{(d)}^{(s)}$. That is, we are interested in selecting the representers output as a whole. Therefore, the first penalty imposed on β's are $\sum_{s=1}^{S} \lambda^{(s)} \sum_{d=1}^{D} \sqrt{r_d} \|\beta_{(d)}^{(s)}\|_2$. Similarly, we can define

$$\tilde{\gamma}_d(\tau) = \sum_{s=1}^{S} \min(1, \frac{\|\beta_{(d)}^{(s)}\|_2}{\tau})$$

and plug into the SIP to improve the integrativeness of representers. For the block-missing problem, we follow the same manner that introduce ℓ_2 penalty and ℓ_2-based SIP to accommodate multivariate representer output.

*Department of Statistics & Data Science, Carnegie Mellon University, Email: qixu@andrew.cmu.edu

†Department of Statistics and Applied Probability, University of California, Santa Barbara, Email: aqu2@ucsb.edu

B Detailed specification of simulation settings

B.1 Simulation settings for R^2 learning

In Section 5.1, covariates $\mathbf{X}^{(s)}$ are generated from $p = 30$ dimensional standard multivariate normal distribution. The representers are generated based on orthogonal bases in $\mathbb{R}^{30}$. Specifically, we first generate a random matrix A where all entries follow standard normal distribution, then QR decomposition is applied to obtain the orthogonal bases, say, $v_1, v_2, \cdots, v_{30}$. The representers are generated as follows:

- $\theta_1(x) = v_1^T x$
- $\theta_2(x) = v_2^T x$
- $\theta_3(x) = v_3^T x$
- $\theta_4(x) = v_4^T x$
- $\theta_5(x) = v_5^T x$
- $\theta_6(x) = v_6^T x$
- $\theta_7(x) = v_7^T x$
- $\theta_8(x) = v_8^T x$
- $\theta_9(x) = v_9^T x$
- $\theta_{10}(x) = v_{10}^T x$
- $\theta_{11}(x) = v_{11}^T \log(1 + x \circ x)$
- $\theta_{12}(x) = v_{12}^T \exp(x)$
- $\theta_{13}(x) = \sum_{i=1}^{15} x_i$
- $\theta_{14}(x) = \sum_{i=16}^{30} x_i$
- $\theta_{15}(x) = v_{15}^T \cos(x)$
- $\theta_{16}(x) = v_{16}^T \tanh(x)$
- $\theta_{17}(x) = v_{17}^T |x|$
- $\theta_{18}(x) = v_{18}^T \sqrt{|x|}$
- $\theta_{19}(x) = v_{19}^T \sqrt[3]{x}$
- $\theta_{20}(x) = v_{20}^T \exp(-x)$
- $\theta_{21}(x) = v_{21}^T \log(1 + |x|)$
- $\theta_{22}(x) = v_{22}^T \sinh(x)$
- $\theta_{23}(x) = v_{23}^T \cosh(x)$
- $\theta_{24}(x) = v_{24}^T \arctan(x)$
- $\theta_{25}(x) = v_{25}^T \arcsin(\text{clip}(x, -1, 1))$
- $\theta_{26}(x) = v_{26}^T \arccos(\text{clip}(x, -1, 1))$
- $\theta_{27}(x) = v_{27}^T \text{clip}(x, -0.999, 0.999)$
- $\theta_{28}(x) = v_{28}^T \text{arcsinh}(x)$
- $\theta_{29}(x) = v_{29}^T \text{arccosh}(\text{clip}(x, 1, \infty))$
- $\theta_{30}(x) = v_{30}^T \max(x, 0.5)$

B.2 Simulation settings for BR^2 learning

In Section 5.2, covariates $\mathbf{X}_m$ are generated from $q = 40$ dimensional multivariate normal distribution with mean zero, and varying covariance matrix. The covariance matrices for 4 modaliteis are as follows: Σ_1 is $q-$dimensional identity matrix, Σ_2 is AR1 matrix with correlation 0.8, Σ_3 is AR1 matrix with correlation 0.5, and Σ_4 is exchangeable matrix with

correlation 0.5.

C Additional simulation studies

C.1 Additional results of R^2 learning

First, we present the simulation results under linear setting, corresponding to the first two distributions in the main text to generate non-zero entries in learners.

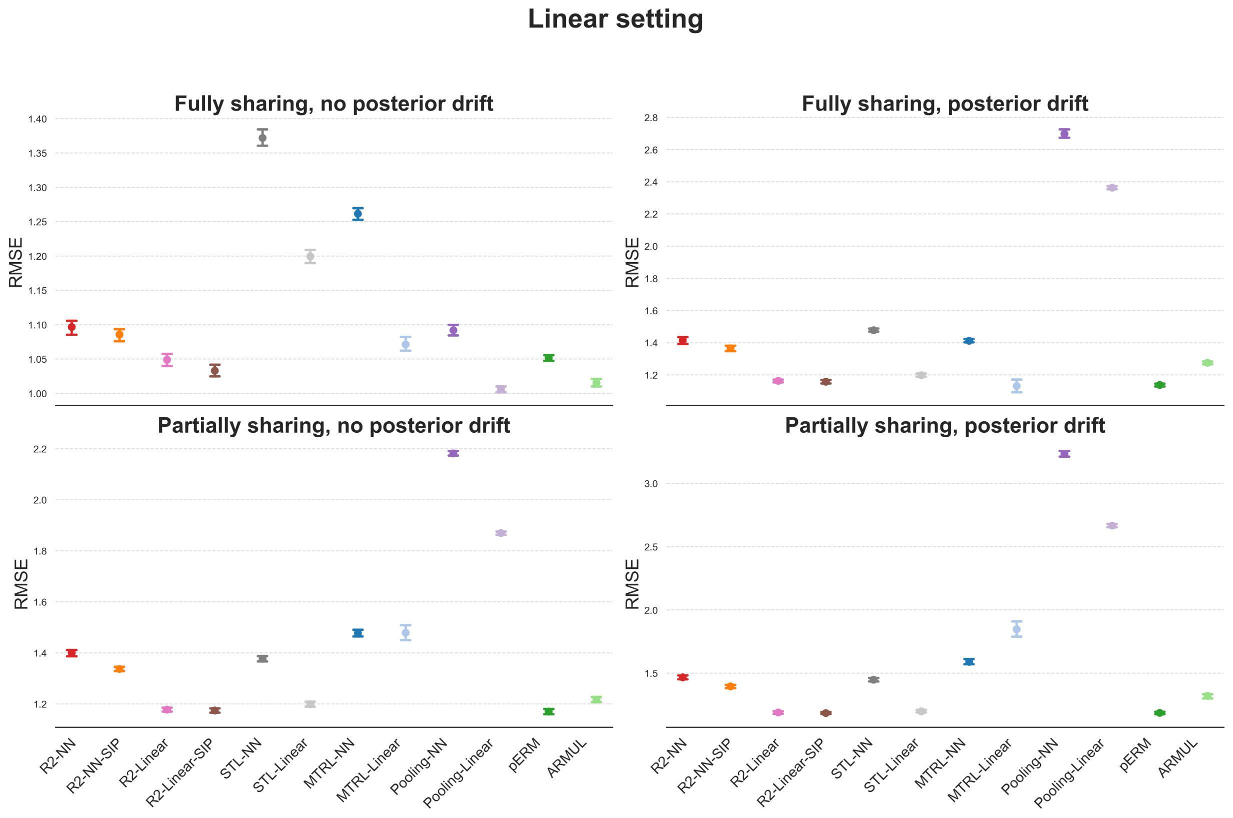

Figure 1: Simulation results for multi-task learning problem under linear setting.

For linear simulation settings, correctly specified models (R2-Linear, R2-Linear-SIP, STL-Linear, MTRL-Linear, Pooling-Linear, pERM and ARMUL) usually perform better than using neural nets as model or representer. In the "fully sharing and no posterior drift" scenario, all data sources share the same distribution. In this scenario, Pooling-

Linear achieves the optimal performance by integrating all data sources. Compared to the STL methods, the MTL methods can effectively integrate information across data sources to improve the predictive performance. Our method with linear representer and SIP achieves comparable results with pERM and ARMUL, which are designed for linear representation. Under the "fully sharing and posterior drift" scenario, the performance of Pooling methods degrade significantly due to posterior drift, and MTRL-Linear stands out as this scenario follows their model specification. Our method, R^2 learning with or without SIP, and pERM all achieve improved predictive performance than STL-Linear, indicating effective data integration. In the "partially sharing and no posterior drift" scenario, our method R^2 learning with or without SIP and pERM again outperforms STL-Linear, while other methods fail to integrate information across data sources. In the "partially sharing and posterior drift" scenario, all competing data integration methods further degrade due to multiple types of heterogeneity, while our R^2 learning methods with linear representers still achieve slight improvement compared to STL-Linear.

Next, we consider the binary classification problem and evaluate the performance of R^2 learning and competing methods. In particular, representers and regression coefficients are generated as in Section 5.1 for regression problem, but generate the response as follows:

$$y_i^{(s)} \sim \text{Bern}\Big\{\frac{1}{1+\exp(-\Theta(\mathbf{x}_i^{(s)})\beta^{(s)})}\Big\}, \quad i = 1, \cdots, 100, s = 1, \cdots, 20.$$

In addition, all competing methods are also applicable for binary classification by adapting binary cross entropy loss. The simulation results are presented as follows.

In the linear setting, R^2 learning with neural net representers is usually superior than R^2 learning with linear representers due to overfitting. Under the fully sharing and no posterior drift scenario, all tasks follow the same distribution, where Pooling-Linear method

achieves the lowest error as expected. All other competing methods outperform STL-Linear, demonstrate different levels of data integration for improved prediction. In the fully sharing and posterior drift scenario, MTRL-Linear is the correctly-specified model, while our R^2 learning with linear representer and SIP, along with pERM achieves the comparable results. In the other two scenarios, MTRL methods perform much worse since it cannot accommodate covariates shift well. Our method and pERM are quite robust to improve prediction performance under these scenarios.

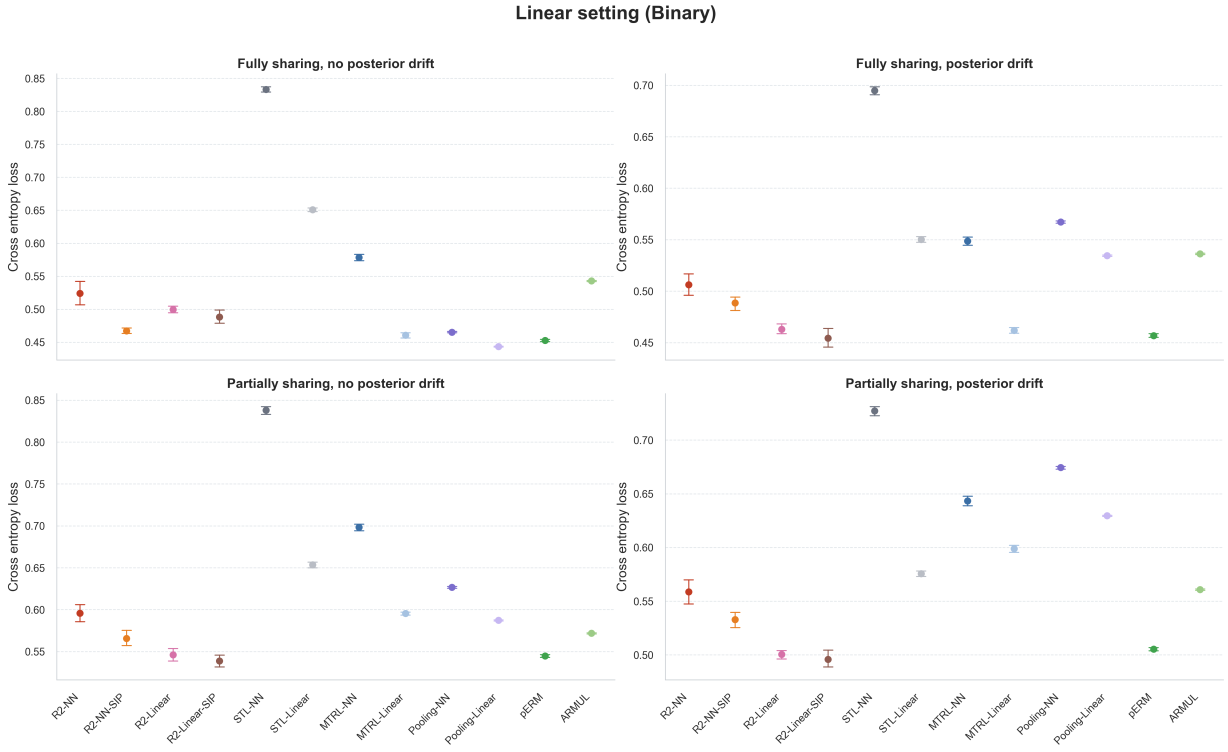

Figure 2: Simulation results for binary classification under linear settings. Y-axis: cross entropy loss (the lower, the better); X-axis: different competing methods.

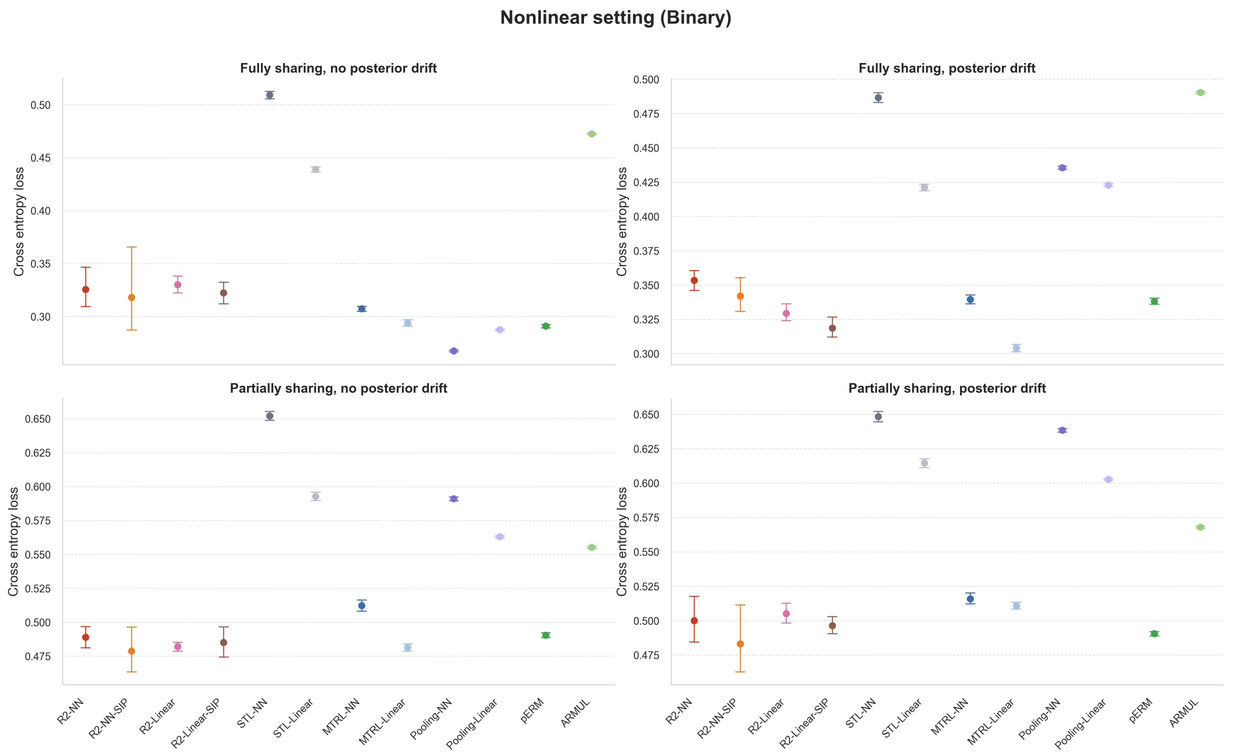

Figure 3: Simulation results for binary classification under nonlinear settings. Y-axis: cross entropy loss (the lower, the better); X-axis: different competing methods.

In the nonlinear setting, Pooling method again achieves the best performance under the fully sharing and no posterior drift scenario, while MTRL still perform well under fully sharing and posterior drift scenario. In the remaining scenarios, R^2 learning with neural net representers and SIP achieves significant improvement over all other competing methods. The advantages of R^2 learning is even greater in the mixed setting, where the underlying data generating process is rather complex.

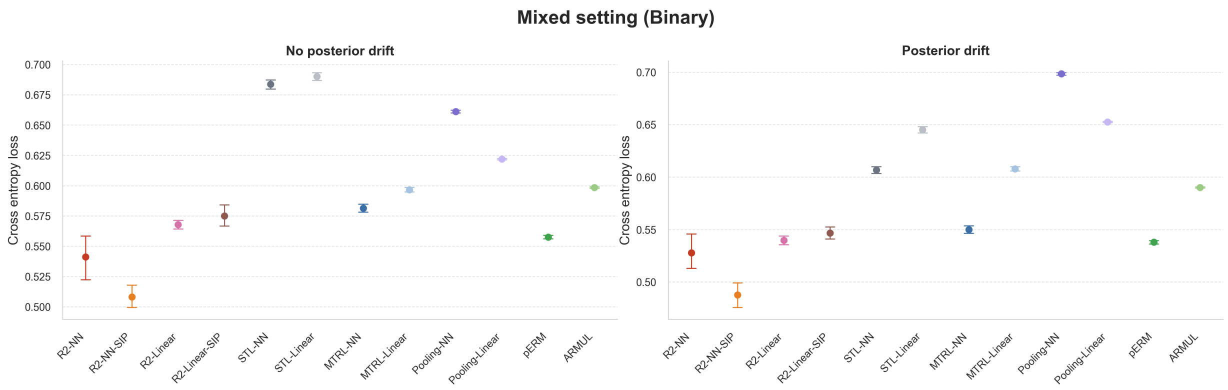

Figure 4: Simulation results for binary classification under mixed settings. Y-axis: cross entropy loss (the lower, the better); X-axis: different competing methods.

In the following, we examine the model performance given varying sample sizes across tasks. In particular, we consider the data generating procedure as in Section 5.1, and the fifth distribution, for 20 tasks. In the first experiment, 100 samples are generated for all 20 tasks; for the second experiment, there are 100 samples for the first 10 tasks and 300 samples for the rest 10 tasks. We validate four setups for R^2 learning: R^2 learning without SIP and neural net representers (R^2-NN in the figure), R^2 learning with SIP and neural net representers (R^2-NN-SIP in the figure), R^2 learning without SIP and linear representer (R^2-Linear in the figure), and R^2 learning with SIP and linear representer (R^2-Linear-SIP in the figure).

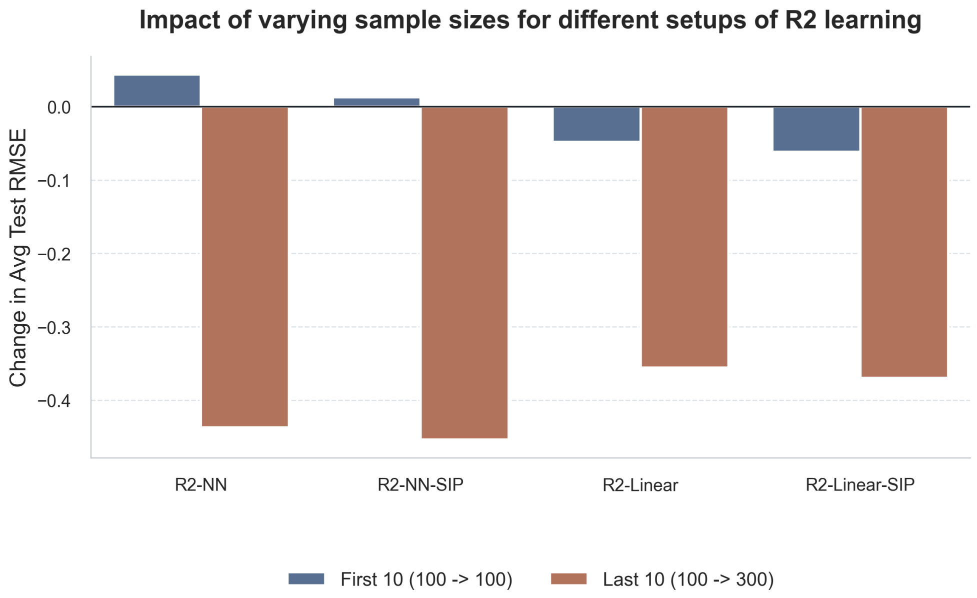

Figure 5: Impact of varying sample sizes for different setups of R^2 learning.

In Figure 5, we exhibit the RMSE change over the first 10 tasks and the last 10 tasks. Apparently, RMSE of the last 10 tasks decrease significantly as sample sizes increase from 100 to 300. In contrast, RMSE of the first 10 tasks changes moderately across different setups of R^2 learning, where some R^2 learning setups improve slightly and other R^2 learning setups worsen the performance minimally. This comparison justifies that our method won't disproportionally favor large sample data sources.

C.2 Additional results of BR^2 learning

In this part, we present additional simulation results for blockwise missing data. In particular, $L = 3$ scenario is presented in the main text for comparing three methods. The $L = 1, 2$ scenarios are shown here.

Specifically, in the $L = 1$ scenario, each modality is only observed by one data source, thereby no information can be borrowed across data sources. As a result, BR^2 learning is slightly worse than STL. However, when L increases, BR^2 learning is able to borrow infor-

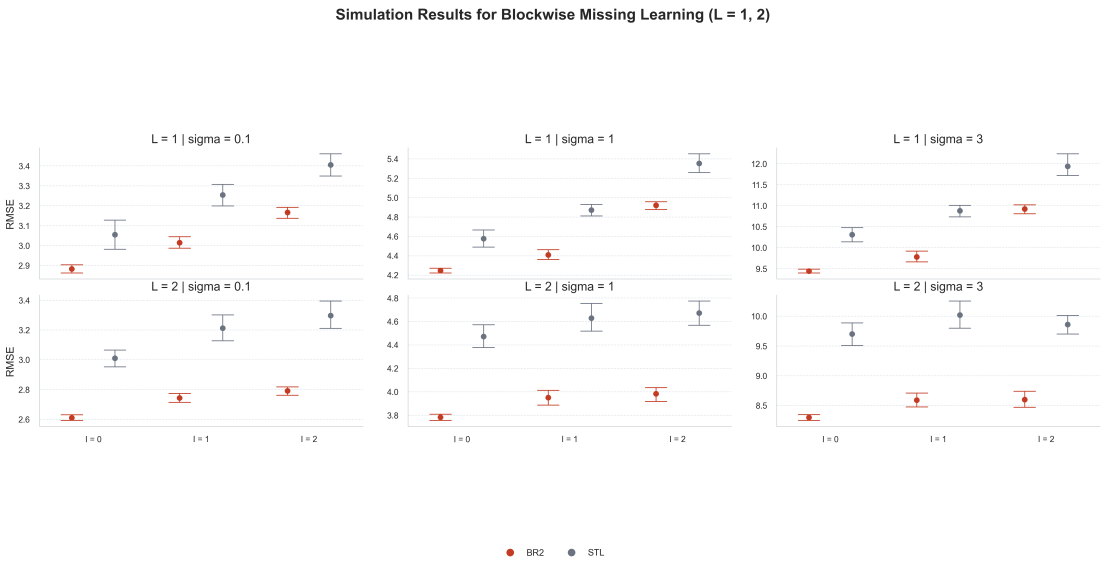

Figure 6: Simulation results for blockwise missing data, $L = 1, 2$ scenario.

mation across data sources and achieve improved prediction accuracy in several scenarios.

D Motivating example

In this subsection, we elaborate the three types of heterogeneity with a concrete example from the Alzheimer's Disease Neuroimaging Initiative* (ADNI) dataset. The data includes four sources, where each source exhibits a different observation pattern. In total, three modalities are collected: magnetic resonance imaging (MRI), positron emission tomography (PET) and gene expression (Gene). The Mini Mental State Examination (MMSE) score is used as a response variable to measure the cognitive impairment of patients.

In Section 6, we aim to build an accurate prediction algorithm to predict the MMSE score given three modalities of covariates. Given the apparent blockwise missingness, existing methods for multi-task learning are not applicable to this dataset. Yu et al. [9], Xue and Qu [8] have analyzed this dataset by assuming a uniform linear model across data sources. In the following, we examine the homogeneity of the marginal distribution of covariates and conditional distribution of responses given covariates across different data sources.

In order to examine the homogeneity of the marginal distribution of each modality, we apply sparse PCA (Witten et al. 7, sparsity is necessary here because the dimension of covariates is greater than sample sizes for all modalities and data sources) to project each modality into 2-dimensional space; and the density of each data source is shown in Figures 7, 8 and 9. The density plots of the MRI modality have apparent discrepancy across the first three data sources, which suggests potential covariates shift.

Next, we examine the posterior homogeneity across four data sources. Since the true models are unknown to us and [9, 8] adopted a linear model for their analysis, we calculate the marginal correlation between each variable and response across four sources, and evaluate whether these marginal correlations are homogeneous across sources. Figure 10 presents the plots of marginal correlations for covariates among three modalities, respectively. In the MRI and Gene modalities, marginal correlations across data sources are quite heterogeneous, where many features exhibit opposite marginal correlation to responses across data sources. Therefore, posterior heterogeneity should be considered when analyzing this dataset.

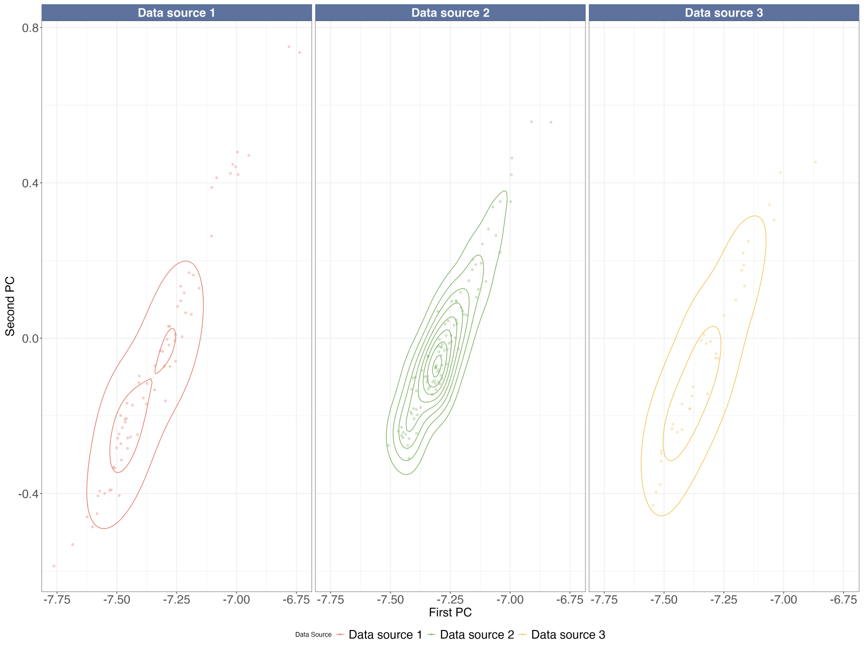

Figure 7: Density plot of 2d projection of data source 1, 2, 3 of MRI modality.

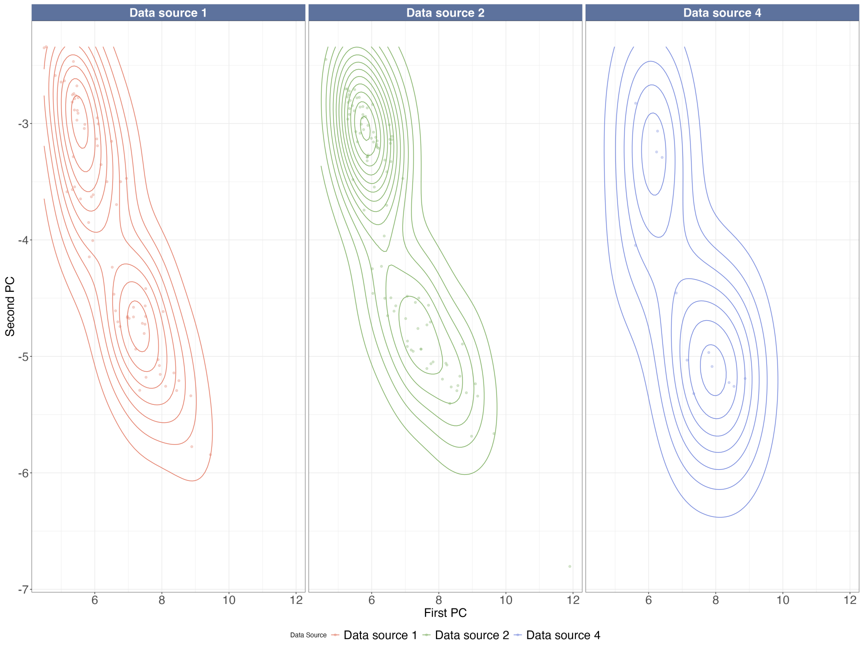

Figure 8: Density plot of 2d projection of data source 1, 2, 4 of PET modality.

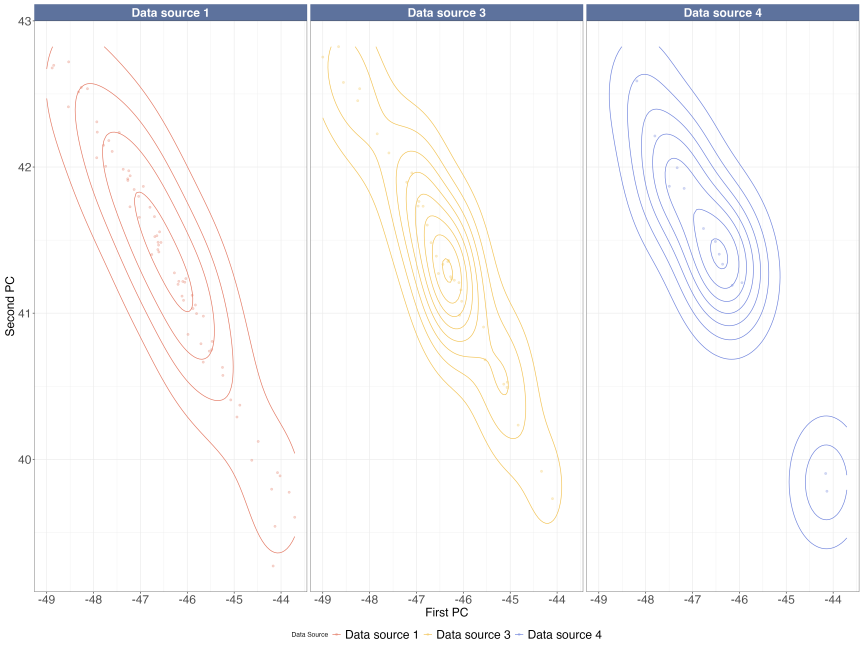

Figure 9: Density plot of 2d projection of data source 1, 3, 4 of Gene modality.

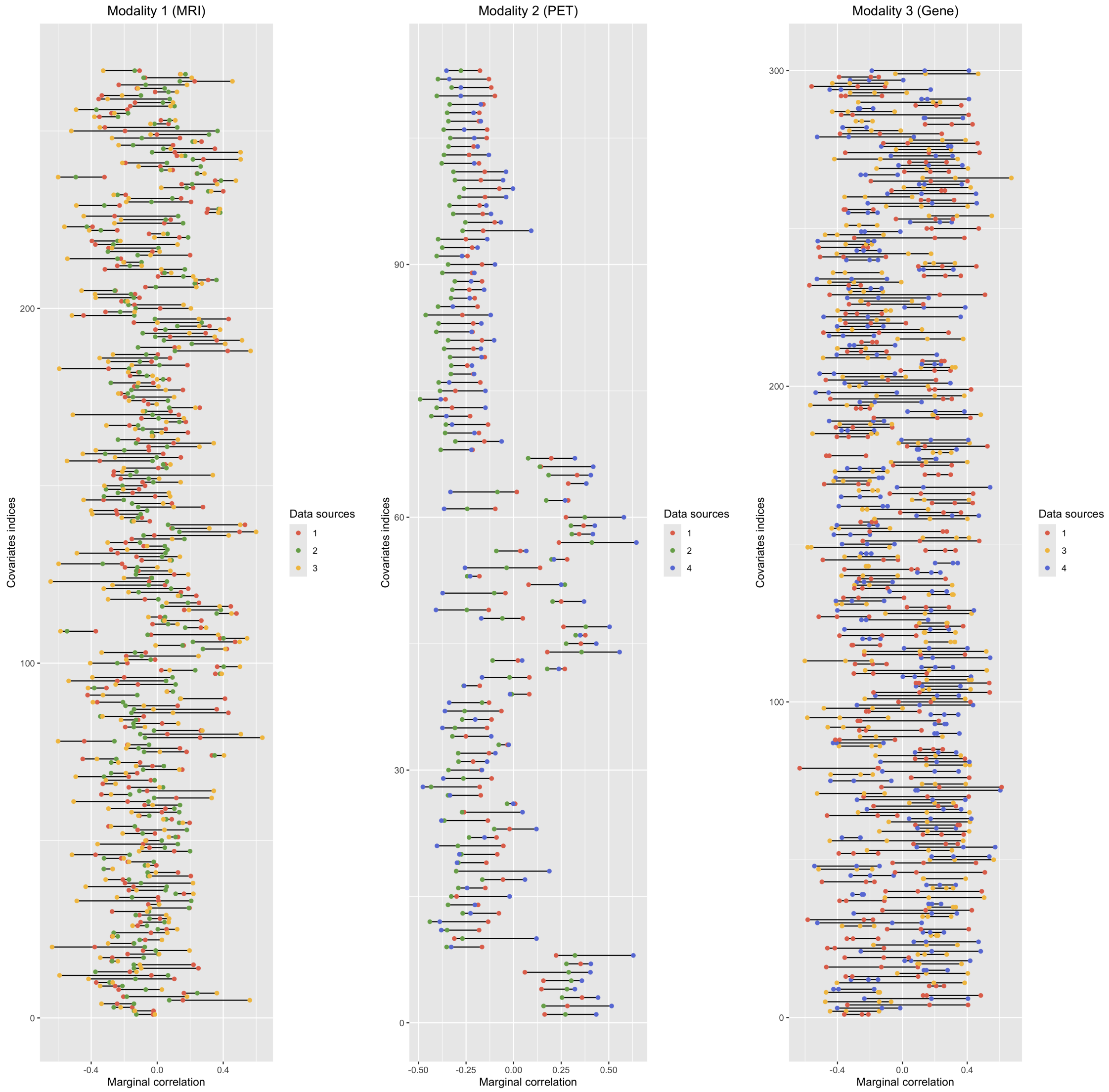

Figure 10: Marginal correlation between each covariates and response across sources. The left plot shows the marginal correlation of covariates in the MRI modality. The central plot shows the marginal correlation of covariates in the PET modality, and the right plot shows the marginal correlation of covariates in the Gene modality.

E Implementation of Competing Methods

In the simulation, pERM is implemented using the open-source code provided by [5] (`https://github.com/ytstat/RL-MTL-TL`), and ARMUL is implemented based on the code from [1] (`https://github.com/kw2934/ARMUL?tab=readme-ov-file`). STL (both linear and neural network versions) and MTRL (both linear and neural network versions)

are implemented in `pytorch`. We adopt default algorithm setups, such as learning rate and epochs, in all the above implementation, and tune the penalty coefficients and neural nets structures in all methods with `optuna`.

F Implementation of Proposed Methods

For both R^2 and BR^2 learning, we implement the optimization using the warmup-plus-alternating procedure. The model is first trained jointly without regularization for a warmup period, so that the representer functions and task-specific regression coefficients can reach a reasonable predictive scale before sparsity is imposed. After warmup, optimization alternates between updating the regression coefficients and updating the representer networks. In the coefficient step, the representers are fixed and the task-specific coefficients are updated under the prediction loss plus the ℓ_1 and SIP penalties, encouraging sparse representer usage and shared support structure across related tasks. In the representer step, the regression coefficients are fixed and the representer parameters are updated to improve data fitting while preserving the current sparse task-representer structure. For R^2, this procedure is applied to multi-task response prediction with task-specific coefficient vectors over shared representers; for BR^2, the same principle is extended to the blockwise-missing setting, where modality/source-specific representers and regressors are trained under the observed block structure. Early stopping is applied after warmup and after at least one full alternating cycle, using validation performance to retain the best model during training.

Three hyperparameters $D, \lambda_1, lambda_2$ play important but different roles: D controls the complexity of representer dictionary, λ_1 controls the sparsity of learners, and λ_2 and τ adjust the representer sharing structure. Our theory result suggests D is not critical for generalization, therefore, a large D (e.g., 30) is chosen in our numerical experiment.

Since λ_1, λ_2 and τ jointly determine the sharing structure of learners across data sources, we suggest to tune them jointly if time and compute permits. If not, we suggest to first tune λ_1 to obtain a good sparse model, and then tune λ_2 and τ jointly to encourage more sharing representers across data sources.

G Pan-Cancer Analysis

In this section, we analyze the pan-cancer data from [4], including five types of cancer: breast cancer (BRCA), melanoma (CM), colorectal cancer (CRC), non-small cell lung cancer (NSCLC), and pancreatic cancer (PDAC). The data were collected from patient-derived xenografts (PDX) experiments, where each PDX line is created through transplanting a tumor into multiple mice, and applying multiple treatments to the mice. Covariates including DNA copy number, gene expression, and mutations were collected for PDX lines. To evaluate the treatment effects, a scaled measure of maximum tumor shrinkage from baseline is collected, termed as best average response (BAR). In our analysis, we are interested in predicting the tumor size shrinkage across five cancer types based on their covariates. Therefore, treatments are ignored and we focus on the untreated mice from each PDX line.

In this dataset, sample sizes for each tumor type are limited: 38 PDX lines for BRCA, 32 PDX lines for CM, 43 PDX lines for CRC, 25 PDX lines for NSCLC, and 35 PDX lines for PDAC. Therefore, the prediction performance of single task learning is inaccurate and highly variable. Furthermore, pan-cancer analysis has shown that many cancer types share some common mechanisms [3], which motivates us to integrate all these five types of tumor to achieve more accurate prediction for all tumor types. Due to the high-dimensionality of covariates, we first apply principal component analysis on each set of covariates: reducing the dimension of DNA copy number from 18,868 to 30, reducing the dimension of RNA

count from 13,577 to 30, and reducing the dimension of mutations from 59 to 10. The obtained low-dimensional representations are treated as covariates and input into predictive algorithms.

To validate the predictive performance, we randomly select 60% PDX lines from each tumor type as training data, an additional 20% PDX lines from each tumor type as validation data for tuning parameters, and the remaining 20% PDX lines from each tumor type as testing data to evaluate the performance. We repeat the sample-splitting 100 times and run all the models on these independent replications. The performance of our R^2 learning and competing methods are evaluated by the root mean squared error (RMSE), reported in Table 1.

Table 1: RMSE (Standard Deviation) for Pan Cancer Data Analysis

**Method**	**BRCA**	**CM**	**CRC**	**NSCLC**	**PDAC**	**Average**
R2-NN-SIP	**1.1067 (0.4219)**	**2.1729 (0.7139)**	**0.9989 (0.2619)**	**1.1354 (0.5533)**	**0.5006 (0.1565)**	**1.3651 (0.2110)**
R2-Linear-SIP	1.7236 (0.5494)	2.5637 (0.8104)	1.2022 (0.3711)	1.7460 (0.5232)	0.8452 (0.1920)	1.7652 (0.2492)
STL-Linear	1.6879 (0.5012)	2.7322 (1.0394)	1.6393 (0.1738)	1.7895 (0.5816)	0.9824 (0.1715)	1.9139 (0.3155)
STL-NN	1.6683 (0.4968)	2.2140 (0.8053)	1.1824 (0.3919)	1.6423 (0.6143)	0.9427 (0.4273)	1.6362 (0.3566)
MTRL-Linear	1.8032 (0.5703)	3.0414 (0.7201)	1.8024 (0.3501)	2.2872 (0.7211)	1.2879 (0.1856)	2.1593 (0.2695)
pERM	1.6376 (0.4618)	2.6506 (0.9177)	1.5650 (0.4719)	2.1232 (0.4522)	1.0629 (0.2431)	1.9261 (0.2691)
ARMUL	1.5936 (0.5037)	2.7626 (0.8067)	1.2699 (0.2355)	1.5028 (0.3730)	0.8610 (0.1283)	1.7595 (0.2925)
Pooling-NN	1.6900 (0.6550)	2.4584 (0.8550)	1.2229 (0.3883)	1.4924 (0.5167)	1.0117 (0.3677)	1.7264 (0.2889)
Pooling-Linear	2.6879 (1.0771)	3.5488 (1.6708)	1.4495 (0.4210)	2.4366 (0.4864)	1.9216 (0.9671)	2.5859 (0.7771)
MTRL-NN	2.9165 (0.9400)	3.2829 (1.2315)	2.2026 (0.8340)	3.1302 (0.8835)	2.3318 (1.0868)	2.9072 (0.4858)

The proposed R^2 learning attains the minimal RMSE for all five types of tumor types, which shows the advantage of our method in integrating heterogeneous datasets. In addition, we analyze the representer retrieval pattern of R^2 learning with and without SIP, to emphasize the effects of SIP. For both models, they all select 12 out of 30 representers. For R^2 learning with SIP, there is one representer shared by three cancer types (BRCA, CM, CRC), and five representers retrieved by two cancer types In contrast, if SIP is not applied, there is just one representer shared by three cancer types (BRCA, CM, CRC), and all other representers are just retrieved once. The average integrativeness of these two models are 19/12 and 14/12, respectively.

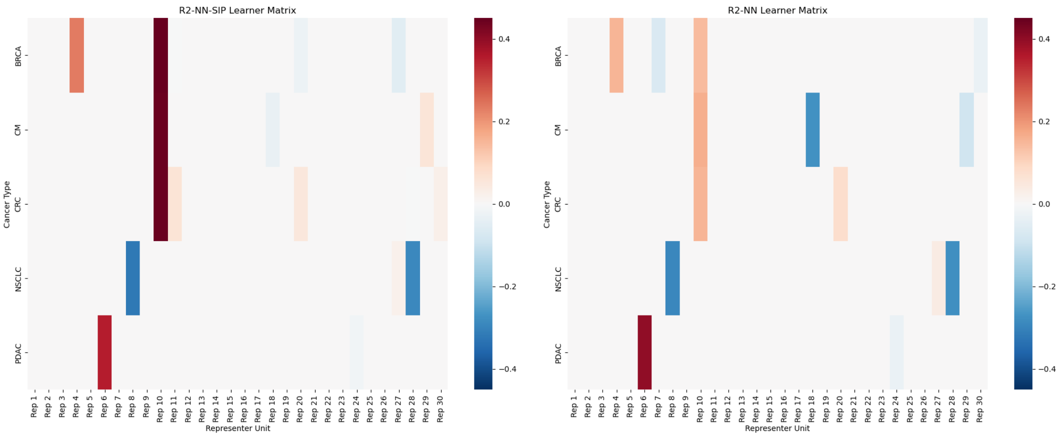

Figure 11: Comparison of learners in the R^2 learning with and without SIP, on the pan-cancer data.

H Technical proofs

In this section, we provide the technical details of the theoretical established in Section 4.

H.1 Proof of Lemma 4.1

Proof. In this section, we quantify the complexity of representers for any fixed β and data points $(\mathbf{x}_i^{(s)}, y_i^{(s)}), i \in [n], s \in [S]$:

$$
\begin{aligned}
G_\beta(\boldsymbol{\sigma}) &= \sup_{\Theta \in \mathcal{F}} \frac{1}{nS} \sum_{s=1}^{S} \sum_{i=1}^{n} \sigma_{s,i} \langle \Theta(\mathbf{x}_i^{(s)}), \beta^{(s)} \rangle \\
&= \sup_{\Theta \in \mathcal{F}} \frac{1}{nS} \sum_{s=1}^{S} \sum_{i=1}^{n} \sigma_{s,i} \sum_{d=1}^{D} \theta_d(\mathbf{x}_i^{(s)}) \beta_d^{(s)}
\end{aligned}
$$

In this Lemma, we provide an upper bound on $\mathbb{E}[G_\beta(\boldsymbol{\sigma})]$. Before we move on, we define $I_d = \{s : \beta_d^{(s)} \neq 0\}$ as the set of tasks retrieving the dth representer, and define $\gamma_d = |I_d|$.

Then we have

$$
\begin{aligned}
\mathbb{E}[G_{\boldsymbol{\sigma}}(\beta)] &= \frac{1}{nS}\mathbb{E}\Big[\sup_{\Theta\in\mathcal{F}}\sum_{s=1}^{S}\sum_{i=1}^{n}\sigma_{s,i}\sum_{d=1}^{D}\theta_d(\mathbf{x}_i^{(s)})\beta_d^{(s)}\Big] \\
&= \frac{1}{nS}\sum_{d:\gamma_d>0}\mathbb{E}\Big[\sup_{\theta_d\in\mathcal{F}_d}\sum_{s\in I_d}\sum_{i=1}^{n}\sigma_{s,i}\theta_d(\mathbf{x}_i)\beta_d^{(s)}\Big] \\
&= \frac{1}{nS}\sum_{d:\gamma_d>0}n\gamma_d\mathbb{E}\Big[\sup_{\theta_d\in\mathcal{F}_d}\frac{1}{n\gamma_d}\sum_{s\in I_d}\sum_{i=1}^{n}\sigma_{s,i}\theta_d(\mathbf{x}_i^{(s)})\beta_d^{(s)}\Big] \\
&\overset{(i)}{\leq} \frac{1}{nS}\sum_{d:\gamma_d>0}n\gamma_d\alpha\mathbb{E}\Big[\sup_{\theta_d\in\mathcal{F}_d}\frac{1}{n\gamma_d}\sum_{s\in I_d}\sum_{i=1}^{n}\sigma_{s,i}\theta_d(\mathbf{x}_i^{(s)})\Big] \\
&= \frac{\alpha}{S}\sum_{d:\gamma_d>0}\gamma_d\hat{\mathfrak{R}}_{n\gamma_d}(\mathcal{G}_d)
\end{aligned}
$$

where (i) holds since $|\beta_d^{(s)}| \leq \|\beta^{(s)}\|_1 \leq \alpha$. Additionally, due to the symmetric of $\mathcal{G}_d$ (if $\theta_d \in \mathcal{G}_d$, then $-\theta_d$ also belongs to $\mathcal{G}_d$), $|\beta_d^{(s)}|$ can be pulled out by taking the supremum over a symmetric function class because of contraction lemma [2]. $\hat{\mathfrak{R}}_{n\gamma_d}(\mathcal{G}_d)$, empirical Rademacher complexity of function class $\mathcal{G}_d$, measures the complexity of function class $\mathcal{G}_d$, and sample size $n\gamma_d$ is the number of total samples used to learn θ_d in the R^2 learning. For many commonly adopted function classes, $\mathfrak{R}_n(\mathcal{G}) \leq O(\sqrt{\frac{C_\mathcal{G}}{n}})$ with constant $C_\mathcal{G}$ associated with function class $\mathcal{G}$. For example, $\mathfrak{R}_n(\mathcal{G}) \leq O(\sqrt{\frac{B^2X^2}{n}})$ for norm-constrained linear space $\mathcal{G} = \{g(\mathbf{x}) = \langle \mathbf{w}, \mathbf{x}\rangle : \|\mathbf{w}\| \leq B\}$ for $\{\mathbf{x} : \|\mathbf{x}\| \leq X\}$. In addition, for a function class of two-layer feed-forward neural nets $\mathcal{G} = \{g_W(x) = W_2\sigma(W_1x) \mid \|W_1\|_F \leq B_1, \|W_2\|_F \leq B_2\}$, its Rademacher complexity scales as $O(\frac{B_1B_2}{\sqrt{n}})$. Therefore, in the following derivation, we let $\hat{\mathfrak{R}}_{n\gamma_d}(\mathcal{G}_d) \leq \sqrt{\frac{C_d}{n\gamma_d}}$ where C_d is a constant dependent on the choice of function class $\mathcal{G}_d$. Further, $C_\Theta \geq C_d$ for $d \in [D]$ is a uniform upper bound constant for all representers. Then

we have

$$
\begin{aligned}
\mathbb{E}[G_\beta(\boldsymbol{\sigma})] &\leq \frac{\alpha}{S} \sum_{d:\gamma_d>0} \gamma_d \hat{\mathfrak{R}}_{n\gamma_d}(\mathcal{F}_d) \\
&\leq \frac{\alpha}{S} \sum_{d:\gamma_d>0} \gamma_d \sqrt{\frac{C_d}{n\gamma_d}} \\
&= \frac{\alpha}{S} \sqrt{\frac{C_\Theta}{n}} \sum_{d=1}^{D} \sqrt{\gamma_d}
\end{aligned}
$$

□

H.2 Lemma G.1

Lemma H.1. *For any fixed β and data realizations, we have the following concentration inequality:*

$$
\mathbb{P}\Big(G_\beta(\boldsymbol{\sigma}) - \mathbb{E}[G_\beta(\boldsymbol{\sigma})] \geq \epsilon \Big) \leq \exp(-\frac{nS\epsilon^2}{2B^2\alpha^2}).
$$

Proof. Note that $G_\beta(\boldsymbol{\sigma})$ is viewed as a function of Rademacher random variables, we may change one value of $\sigma_{s,i}$ to $-\sigma_{s,i}$, denoted as $\sigma'_{s,i}$ and denote $(\sigma_{1,1}, \cdots, \sigma'_{s,i}, \cdots, \sigma_{S,n})$ as $\boldsymbol{\sigma}'$. Then we have the following inequality to bound the differences for any s, i:

$$
\begin{aligned}
|G_\beta(\boldsymbol{\sigma}) - G_\beta(\boldsymbol{\sigma}')| &\leq \frac{2}{nS} \sup_{\theta \in \mathcal{F}} \sum_{d=1}^{D} |\theta_d(\mathbf{x}_i^{(s)})\beta_d^{(s)}| \\
&\leq \frac{2}{nS} B \sum_{d=1}^{D} |\beta_d^{(s)}| = \frac{2}{nS} B\alpha
\end{aligned}
$$

Followed by the McDiarmid's inequality [6], we obtain the expected concentration inequality. □

H.3 Proof of Theorem 4.1

Proof. First of all, the excess risk bound can be decomposed as follows:

$$\mathcal{R}(\hat{\Theta}, \hat{\beta}) - \mathcal{R}(\Theta^*, \beta^*) = \underbrace{\mathcal{R}(\hat{\Theta}, \hat{\beta}) - \hat{\mathcal{R}}(\hat{\Theta}, \hat{\beta})}_{\mathcal{E}_1} + \underbrace{\hat{\mathcal{R}}(\hat{\Theta}, \hat{\beta}) - \hat{\mathcal{R}}(\Theta^*, \beta^*)}_{\mathcal{E}_2} + \underbrace{\hat{\mathcal{R}}(\Theta^*, \beta^*) - \mathcal{R}(\Theta^*, \beta^*)}_{\mathcal{E}_3},$$

where $\mathcal{E}_2 \leq 0$ by the definition of empirical risk minimizer. Since $|\mathcal{E}_1|, |\mathcal{E}_3| \leq \sup_{\Theta,\beta} |\hat{\mathcal{R}}(\Theta, \beta) - \mathcal{R}(\Theta, \beta)|$, we can obtain the excess risk bound by the following uniform convergence statement:

$$\mathbb{P}(\mathcal{R}(\hat{\Theta}, \hat{\beta}) - \mathcal{R}(\Theta^*, \beta^*) \geq \epsilon) \leq \mathbb{P}(\sup_{\Theta,\beta} |\hat{\mathcal{R}}(\Theta, \beta) - \mathcal{R}(\Theta, \beta)| \geq \epsilon/2).$$

We define $G_{nS} = \sup_{\Theta,\beta} \mathcal{R}(\Theta, \beta) - \hat{\mathcal{R}}(\Theta, \beta)$, which is a random variable dependent on nS samples $(\mathbf{x}_i^{(s)}, y_i^{(s)})$ for $i \in [n]$ and $s \in [S]$, and $G'_{nS} = \sup_{\Theta,\beta} \hat{\mathcal{R}}(\Theta, \beta) - \mathcal{R}(\Theta, \beta)$, then we have

$$\mathbb{P}(\sup_{\Theta,\beta} |\hat{\mathcal{R}}(\Theta, \beta) - \mathcal{R}(\Theta, \beta)| \geq \epsilon/2) \leq \mathbb{P}(G_{nS} \geq \epsilon/2) + \mathbb{P}(G'_{nS} \geq \epsilon/2).$$

Since G_{nS} and G'_{nS} are sample mean of loss function on i.i.d samples from $\mu^{(s)}$ and B-boundedness of the loss function, we may apply McDiarmid's inequality [6] to obtain the following concentration inequality for G_{nS}:

$$\mathbb{P}(G_{nS} - \mathbb{E}[G_{nS}] \geq \epsilon) \leq \exp(-2n\epsilon^2/B^2),$$

and based on the definition of Rademacher complexity, $\mathbb{E}[G_{nS}] \leq 2\mathfrak{R}_{nS}[\mathcal{A}]$ where $\mathcal{A} = \{(\mathbf{x}^{(s)}, y^{(s)}) \mapsto \ell(y^{(s)}, \sum_{d=1}^{D} \theta_d(\mathbf{x}^{(s)})\beta_d^{(s)}), s \in [S], \theta_d \in \mathcal{F}_d, \beta^{(s)} \in \mathcal{H}^{(s)}\}$. By the $L-$Lipschitz

condition for the first entry given any second entry of $\ell(\cdot,\cdot)$, we have $\mathfrak{R}_{nS}[\mathcal{A}] \le L\mathfrak{R}_{nS}[\mathcal{G}\circ\mathcal{H}]$ based on contraction principal [2] where $\mathcal{G}\circ\mathcal{H}$ denotes the function class considered in R^2 learning for all tasks. Combining all above results, we have

$$\mathcal{R}(\hat{\Theta},\hat{\beta}) - \mathcal{R}(\Theta^*,\beta^*) \le 4L\mathfrak{R}_{nS}[\mathcal{G}\circ\mathcal{H}] + \sqrt{\frac{2B^2\log(2/\delta)}{n}},$$

with probability at least $1-\delta$. Therefore, all we need to finish is the upper bound of the Rademacher complexity of the function class considered in the R^2 learning. First of all, we define the empirical Rademacher complexity, whose upper bound will be established:

$$\hat{\mathfrak{R}}_{nS}[\mathcal{G}\circ\mathcal{H}] = \mathbb{E}_{\boldsymbol{\sigma}}\left[\sup_{\Theta\in\mathcal{G},\beta\in\mathcal{H}} \frac{1}{nS}\sum_{s=1}^{S}\sum_{i=1}^{n}\sigma_{s,i}\langle\Theta(\mathbf{x}_i^{(s)}),\beta^{(s)}\rangle\middle|(\mathbf{x}_i^{(s)},y_i^{(s)}), i\in[n], s\in[S]\right],$$

where $\sigma_{s,i}$ are i.i.d Rademacher random variables. Based on the definition of $G_\beta(\boldsymbol{\sigma})$ in the proof of Lemma 4.1, the empirical Rademacher complexity is equivalent to $\mathbb{E}_{\boldsymbol{\sigma}}\left[\sup_{\beta\in\mathcal{H}} G_\beta(\boldsymbol{\sigma})\right]$ for fixed realization of $(\mathbf{x}_i^{(s)},y_i^{(s)}), i\in[n], s\in[S]$. Due to the structure of $\mathcal{H}$, we can have

the following derivations:

$$
\begin{aligned}
\mathbb{E}_{\boldsymbol{\sigma}}\left[\sup_{\beta\in\mathcal{H}} G_\beta(\boldsymbol{\sigma})\right] &\leq \mathbb{E}_{\boldsymbol{\sigma}}\left[\sup_{\beta\in\mathrm{conv}(\mathcal{H})} G_\beta(\boldsymbol{\sigma})\right] \\
&\overset{(i)}{=} \mathbb{E}_{\boldsymbol{\sigma}}\left[\max_{\beta\in\mathrm{ext}(\mathrm{conv}(\mathcal{H}))} G_\beta(\boldsymbol{\sigma})\right] \\
&= \int_0^\infty \mathbb{P}(\max_{\beta\in\mathrm{ext}(\mathrm{conv}(\mathcal{H}))} G_\beta(\boldsymbol{\sigma}) \geq t)dt \\
&= \int_0^{\mathbb{E}_{\boldsymbol{\sigma}} G_\beta(\boldsymbol{\sigma})+\delta} \mathbb{P}(\max_{\beta\in\mathrm{ext}(\mathrm{conv}(\mathcal{H}))} G_\beta(\boldsymbol{\sigma}) \geq t)dt \\
&+ \int_{\mathbb{E}_{\boldsymbol{\sigma}} G_\beta(\boldsymbol{\sigma})+\delta}^\infty \mathbb{P}(\max_{\beta\in\mathrm{ext}(\mathrm{conv}(\mathcal{H}))} G_\beta(\boldsymbol{\sigma}) \geq t)dt \\
&\leq \mathbb{E}_{\boldsymbol{\sigma}} G_\beta(\boldsymbol{\sigma}) + \delta + \sum_{\beta\in\mathrm{ext}(\mathrm{conv}(\mathcal{H}))} \int_\delta^\infty \mathbb{P}(G_\beta(\boldsymbol{\sigma}) - \mathbb{E}_{\boldsymbol{\sigma}} G_\beta(\boldsymbol{\sigma}) \geq t)dt \\
&\leq \sup_{\beta\in\mathcal{H}} \mathbb{E}_{\boldsymbol{\sigma}} G_\beta(\boldsymbol{\sigma}) + \delta + (2D)^S \int_\delta^\infty \exp(-\frac{nSt^2}{2B^2\alpha^2})dt \\
&\leq \sup_{\beta\in\mathcal{H}} \mathbb{E}_{\boldsymbol{\sigma}} G_\beta(\boldsymbol{\sigma}) + \delta + (2D)^S \frac{\exp(-\frac{nS\delta^2}{2B^2\alpha^2})}{\frac{nS\delta}{B^2\alpha^2}}.
\end{aligned}
$$

Note that $\mathrm{conv}(\mathcal{H}) = \{(\beta^{(1)}, \cdots, \beta^{(S)}) \mid \beta^{(1)} \in \mathcal{H}^{(s)}\}$ and $\mathrm{ext}(\mathrm{conv}(\mathcal{H}))$ contains all sparse coordinates in S sources with cardnality $(2D)^S$. The equation (i) holds for linear functions on convex compact sets. By letting $\delta = \sqrt{\frac{2B^2\alpha^2\log(2D)}{n}}$, we can conclude the results in Theorem 4.1. □

H.4 Proof of Corollary 4.1

Proof. In Corollary 4.1, we consider the regression coefficient space $\mathcal{H}_{\eta,\tau}$, which is the subset of $\mathcal{H}$. Based on the definition of SIP, we revisit the upper bound for $\mathbb{E}_{G_{\boldsymbol{\sigma}}}(\beta)$ for any fixed $\beta \in \mathcal{H}_{\eta,\tau}$.

In the following, we establish the upper bound with respect to $\tilde{\gamma}_d$'s. Let's define $g_d^{(s)}(\tau) =$

$\mathbb{I}(0 < |\beta_d^{(s)}| < \tau)$ as the upper bound of the approximation error:

$$\gamma_d - \tilde{\gamma}_d(\tau) \leq \sum_{s=1}^{S} g_d^{(s)}(\tau).$$

Now, let's reconsider the quantity of interest as follows:

$$\begin{aligned}
\mathbb{E}[G_{\boldsymbol{\sigma}}(\beta)] &= \frac{1}{nS}\mathbb{E}\Big[\sup_{\Theta\in\mathcal{F}}\sum_{s,i}\sigma_{s,i}\sum_{d}\theta_d(\mathbf{x}_i^{(s)})\beta_d^{(s)}\Big] \\
&\leq \frac{1}{nS}\sum_{d=1}^{D}\mathbb{E}\Big[\sup_{\theta_d\in\mathcal{F}_d}\sum_{s\in I_d}|\beta_d^{(s)}|\sum_{i}\sigma_{s,i}\theta_d(\mathbf{x}_i^{(s)})\Big] \\
&\leq \frac{1}{nS}\sum_{d=1}^{D}\mathbb{E}\Big[\sup_{\theta_d\in\mathcal{F}_d}\sum_{s\in I_d}[\alpha(1-g_d^{(s)})+\tau g_d^{(s)}]\sum_{i}\sigma_{s,i}\theta_d(\mathbf{x}_i^{(s)})\Big] \\
&\leq \underbrace{\frac{1}{nS}\sum_{d=1}^{D}\alpha\mathbb{E}\Big[\sup_{\theta_d\in\mathcal{F}_d}\sum_{s\in I_d}(1-g_d^{(s)}(\tau))\sum_{i}\sigma_{s,i}\theta_d(\mathbf{x}_i^{(s)})\Big]}_{A} \\
&\quad + \underbrace{\frac{1}{nS}\sum_{d=1}^{D}\tau\mathbb{E}\Big[\sup_{\theta_d\in\mathcal{F}_d}\sum_{s\in I_d}g_d^{(s)}(\tau)\sum_{i}\sigma_{s,i}\theta_d(\mathbf{x}_i^{(s)})\Big]}_{B}.
\end{aligned}$$

Now let's define the set $I_{d,1} = \{s : s \in I_d, g_d^{(s)}(\tau) = 0\}$ as the data sources whose dth coordinate $|\beta_d^{(s)}| \geq \tau$ and $I_{d,2} = \{s : s \in I_d, g_d^{(s)}(\tau) = 1\}$ as the data sources whose dth coordinate $0 < |\beta_d^{(s)}| < \tau$. Then we have

$$\begin{aligned}
A &\leq \frac{\alpha}{nS}\sum_{d:|I_{d,1}|>0} n|I_{d,1}|\mathbb{E}\Big[\sup_{\theta_d\in\mathcal{F}_d}\frac{1}{n|I_{d,1}|}\sum_{s\in I_{d,1}}\sum_{i}\sigma_{s,i}\theta_d(\mathbf{x}_i^{(s)})\Big] \\
&\leq \frac{\alpha}{nS}\sum_{d:|I_{d,1}|>0} n|I_{d,1}|\sqrt{\frac{C_\Theta}{n|I_{d,1}|}} \leq \frac{\alpha}{S}\sqrt{\frac{C_\Theta}{n}}\sum_{d=1}^{D}\sqrt{|I_{d,1}|} \\
&\leq \frac{\alpha}{S}\sqrt{\frac{C_\Theta}{n}}\sum_{d=1}^{D}\sqrt{\tilde{\gamma}_d(\tau)},
\end{aligned}$$

where the last inequality holds because $|I_{d,1}| = \gamma_d - \sum_{s=1}^{S} g_d^{(s)}(\tau) \leq \tilde{\gamma}_d(\tau)$. In the same

manner, we have

$$B \leq \frac{\tau}{S}\sqrt{\frac{C_\Theta}{n}} \sum_{d:|I_{d,2}|>0} \sqrt{|I_{d,2}|} = \frac{\tau}{S}\sqrt{\frac{C_\Theta}{n}} \sum_{d=1}^{D} \sqrt{g_d(\tau)},$$

where $g_d(\tau) = \sum_{s=1}^{S} g_d^{(s)}(\tau)$.

Based on the definition of $g_d(\tau)$, $g_d(\tau) \leq S$ for all d and τ. Further, for $\beta \in \mathcal{H}_{\eta,\tau}$, $\sum_{d=1}^{D} \sqrt{\tilde{\gamma}_d(\tau)} \leq \eta(\tau)$. Combining all these terms leads to the new upper bound for $\mathbb{E}[G_{\boldsymbol{\sigma}}(\beta)]$:

$$\mathbb{E}[G_{\boldsymbol{\sigma}}(\beta)] \leq \frac{\alpha}{S}\sqrt{\frac{C_\Theta}{n}}\eta(\tau) + \tau\sqrt{\frac{C_\Theta D^2}{nS}}.$$

Then, plug this upper bound into the derivation of Theorem 4.1 leads to upper bound (5.1). □

H.5 Proof of Lemma I.1 and Corollary 4.1

Following the similar steps as in the proof of Lemma 4.1, we have the following inequalities for $\mathbb{E}_{\boldsymbol{\sigma}}[G_\beta(\boldsymbol{\sigma})]$:

$$\begin{aligned}
\mathbb{E}_{\boldsymbol{\sigma}}[G_\beta(\boldsymbol{\sigma})] &= \mathbb{E}_{\boldsymbol{\sigma}}\left[\sup_{\Theta\in\mathcal{F}} \frac{1}{nS}\sum_{s=1}^{S}\sum_{i=1}^{n} \sigma_{s,i}\left[\sum_{m=1}^{M} \mathbb{I}_m^{(s)} \sum_{d=1}^{D} \theta_{m;d}(\mathbf{x}_{i;m}^{(s)})\beta_{m;d}^{(s)}\right]\right] \\
&\leq \frac{1}{nS}\sum_{m=1}^{M}\sum_{d=1}^{D} \mathbb{E}_{\boldsymbol{\sigma}}\left[\sup_{\theta_{m;d}} \sum_{s\in I_{m;d}} |\beta_{m;d}^{(s)}| \sum_i \sigma_{s,i}\theta_{m;d}(\mathbf{x}_{i;m}^{(s)})\right] \\
&\leq \frac{1}{nS}\sum_{m=1}^{M}\sum_{d=1}^{D} \alpha\mathbb{E}\left[\sup_{\theta_{d;m}} \sum_{s\in I_{m;d}} (1 - g_{m;d}^{(s)}(\tau_m)) \sum_i \sigma_{s,i}\theta_{m;d}(\mathbf{x}_{i;m}^{(s)})\right] \\
&+ \frac{1}{nS}\sum_{m=1}^{M}\sum_{d=1}^{D} \tau_m\mathbb{E}\left[\sup_{\theta_{d;m}} \sum_{s\in I_{m;d}} g_{m;d}^{(s)}(\tau_m) \sum_i \sigma_{s,i}\theta_{m;d}(\mathbf{x}_{i;m}^{(s)})\right].
\end{aligned}$$

By the same reasoning as in the proof of Lemma 4.1, we can obtain the desired upper bounds. And the excess risk bound in Corollary 4.1 can be derived based on this results and techniques in the proof of Theorem 4.1.

I Excess risk bound for BR^2 learning

In this subsection, we analyze the generalization error of the BR^2 learning in the block-missing data. Similar to the R^2 learning, here is our postulated function class for approximating $f^{(s)}$:

$$f^{(s)}(\mathbf{X}^{(s)}) = \sum_{m=1}^{M} \mathbb{I}_m^{(s)} \sum_{d=1}^{D} \theta_{m;d}(\mathbf{X}_m^{(s)})\beta_{m;d}^{(s)}, s \in [S],$$

where $\theta_{m;d} \in \mathcal{G}_{m;d}$ of certain function class, and $\Theta \in \mathcal{G} = \{(\theta_{1;1}, \cdots, \theta_{m;d}, \cdots, \theta_{M;D}), \theta_{m;d} \in \mathcal{G}_{m;d}\}$ and $\beta_m^{(s)} = (\beta_{1;m}^{(s)}, \cdots, \beta_{D;m}^{(s)}) \in \mathcal{H}_m^{(s)} = \{\beta \in \mathbb{R}^D, \|\beta\|_1 \le \alpha_m^{(s)}\}$ for $s \in [S]$ and $m \in [M]$. Considering the SIP, the function class for β_m is $\mathcal{H}_m = \{\beta_m^{(s)} \mid s \in \mathcal{O}_m, \beta_m^{(s)} \in \mathcal{H}_m^{(s)}, \sum_{d=1}^{D} \sqrt{\tilde{\gamma}_{m;d}(\tau_m)} \le \eta_m(\tau_m)\}$. For notational brevity, let each modality-associated dictionary Θ_m contain D representers uniformly. We define the empirical risk as follows:

$$\hat{\mathcal{R}}(\Theta, \beta) = \frac{1}{S}\sum_{s=1}^{S} \frac{1}{n}\sum_{i=1}^{n} \ell\Big\{y_i^{(s)}, \sum_{m=1}^{M} \mathbb{I}_m^{(s)} \sum_{d=1}^{D} \theta_{m;d}(\mathbf{x}_{i;m}^{(s)})\beta_{m;d}^{(s)}\Big\},$$

with empirical risk minimizer defined as $\hat{\Theta}$ and $\hat{\beta}$. Analogously, population risk is defined with expectation over $(\mathbf{X}^{(s)}, \mathbf{y}^{(s)}) \sim \mu^{(s)}$:

$$\mathcal{R}(\Theta, \beta) = \frac{1}{S}\sum_{s=1}^{S} \mathbb{E}_{(\mathbf{X}^{(s)}, \mathbf{y}^{(s)}) \sim \mu^{(s)}} \left[\ell\Big\{\mathbf{y}^{(s)}, \sum_{m=1}^{M} \mathbb{I}_m^{(s)} \sum_{d=1}^{D} \theta_{m;d}(\mathbf{X}_m^{(s)})\beta_{m;d}^{(s)}\Big\}\right].$$

The missing indicators, $\mathbb{I}_m^{(s)}$, are embedded in both $\hat{\mathcal{R}}(\Theta, \beta)$ and $\mathcal{R}(\Theta, \beta)$. Note that learning $f^{(s)}$ using only observed modalities introduces non-negligible approximation errors. Since our primary interest lies in whether integrating multi-source data outperforms individual learning despite missing blocks, we focus on analyzing the generalization error with inclusion of $\mathbb{I}_m^{(s)}$. In the following exposition, we denote the population risk minimizer as Θ^* and β^*. And we are interested in the estimation error $\mathcal{R}(\hat{\Theta}, \hat{\beta}) - \mathcal{R}(\Theta^*, \beta^*)$.

Following the same steps as in Lemma 4.1, we obtain an upper bound on the complexity of learning the representation dictionary.

Lemma I.1. *For any $\beta_m \in \mathcal{H}_m$ and fixed realizations $(\mathbf{x}_i^{(s)}, y_i^{(s)})_{i=1}^n, s \in [S]$, we define*

$$\mathbf{G}_\beta(\boldsymbol{\sigma}) = \sup_{\Theta \in \mathcal{F}} \frac{1}{nS} \sum_{s=1}^{S} \sum_{i=1}^{n} \sigma_{s,i} \left[\sum_{m=1}^{M} \mathbb{I}_m^{(s)} \sum_{d=1}^{D} \theta_{m;d}(\mathbf{x}_{i;m}^{(s)}) \beta_{m;d}^{(s)} \right],$$

where $\sigma_{s,i}$ are i.i.d Rademacher random variables, then we have:

$$\mathbb{E}_{\boldsymbol{\sigma}} \mathbf{G}_\beta(\boldsymbol{\sigma}) \leq \sum_{m=1}^{M} \frac{\alpha_m}{S} \sqrt{\frac{C_{\Theta_m}}{n}} \eta_m(\tau_m) + \sum_{m=1}^{M} \tau_m \sqrt{\frac{C_{\Theta_m} D^2}{nS}}, \tag{1}$$

where $\alpha_m = \max_s \alpha_m^{(s)}$. The constant C_{Θ_m} is the uniform upper bound $C_{\Theta_m} \geq C_{\theta_{m;d}}$ for all $d \in [D]$, where $\mathbb{E}\left\{ \sup_{\theta_{m;d} \in \mathcal{F}_{m;d}} \frac{1}{n} \sum_{i=1}^{n} \sigma_i \theta_{m;d}(\mathbf{x}_i) \right\} \lesssim \sqrt{\frac{C_{\theta_{m;d}}}{n}}$.

Lemma I.1 follows all properties derived in Lemma 4.1. Since SIP is applied to each modality, varying values of γ_m and $\lambda_{m;2}$ could be selected for different modalities in (3.8). Therefore, the upper bound in (1) aggregates the complexities of each modality dictionary with modality-specific values of $\eta_m(\tau_m)$. In the same manner, we can derive the excess risk bound for the BR^2 learning as shown in the following corollary.

Corollary I.1. *Suppose Assumption 4.1 holds, and the dictionary size is fixed as D for all modalities. Let $(\mathbf{x}_i^{(s)}, y_i^{(s)})$ be identically and independently distributed random samples*

following distribution $\mu^{(s)}$ defined over $\mathcal{X} \times \mathcal{Y}$, then for empirical risk minimizer $\hat{\Theta}$ and $\hat{\beta}$, with probability $1-\delta$, we have

$$\mathcal{R}(\hat{\Theta}, \hat{\beta}) - \mathcal{R}(\Theta^*, \beta^*) \leq 4L \sum_{m=1}^{M} \frac{\alpha_m}{S} \sqrt{\frac{C_{\Theta_m}}{n}} \eta_m(\tau_m) + 4L \sum_{m=1}^{M} \tau_m \sqrt{\frac{C_{\Theta_m} D^2}{nS}} + 4\alpha BLM \sqrt{\frac{2\log(2D)}{n}} + B\sqrt{\frac{2\log(2/\delta)}{n}}.$$

References

[1] Duan, Y. and Wang, K. (2023). Adaptive and robust multi-task learning. *The Annals of Statistics*, 51(5):2015–2039.

[2] Ledoux, M. and Talagrand, M. (2013). *Probability in Banach Spaces: isoperimetry and processes*. Springer Science & Business Media.

[3] Petralia, F., Ma, W., Yaron, T. M., Caruso, F. P., Tignor, N., Wang, J. M., Charytonowicz, D., Johnson, J. L., Huntsman, E. M., Marino, G. B., et al. (2024). Pan-cancer proteogenomics characterization of tumor immunity. *Cell*, 187(5):1255–1277.

[4] Rashid, N. U., Luckett, D. J., Chen, J., Lawson, M. T., Wang, L., Zhang, Y., Laber, E. B., Liu, Y., Yeh, J. J., Zeng, D., et al. (2020). High-dimensional precision medicine from patient-derived xenografts. *Journal of the American Statistical Association*, 116(535):1140–1154.

[5] Tian, Y., Gu, Y., and Feng, Y. (2023). Learning from similar linear representations: Adaptivity, minimaxity, and robustness. *arXiv preprint arXiv:2303.17765*.

[6] Wainwright, M. J. (2019). *High-dimensional statistics: A non-asymptotic viewpoint*, volume 48. Cambridge university press.

[7] Witten, D. M., Tibshirani, R., and Hastie, T. (2009). A penalized matrix decomposition,

with applications to sparse principal components and canonical correlation analysis. *Biostatistics*, 10(3):515–534.

[8] Xue, F. and Qu, A. (2021). Integrating multisource block-wise missing data in model selection. *Journal of the American Statistical Association*, 116(536):1914–1927.

[9] Yu, G., Li, Q., Shen, D., and Liu, Y. (2020). Optimal sparse linear prediction for block-missing multi-modality data without imputation. *Journal of the American Statistical Association*, 115(531):1406–1419.
